# Action Quality Assessment Model Using Specialists’ Gaze Location and Kinematics Data—Focusing on Evaluating Figure Skating Jumps

**DOI:** 10.3390/s23229282

**Published:** 2023-11-20

**Authors:** Seiji Hirosawa, Takaaki Kato, Takayoshi Yamashita, Yoshimitsu Aoki

**Affiliations:** 1 Graduate School of Science and Technology, Keio University, Yokohama 223-8522, Japan; 2 Faculty of Sport and Health Sciences, Toin University of Yokohama, Yokohama 225-8503, Japan; 3 Faculty of Environment and Information Studies, Keio University, Fujisawa 252-0882, Japan; 4 College of Engineering, Chubu University, Kasugai 487-8501, Japan

**Keywords:** computer vision application, human action evaluation, sports activity scoring, double-axel jump, grade of execution score, sports officials

## Abstract

Action quality assessment (AQA) tasks in computer vision evaluate action quality in videos, and they can be applied to sports for performance evaluation. A typical example of AQA is predicting the final score from a video that captures an entire figure skating program. However, no previous studies have predicted individual jump scores, which are of great interest to competitors because of the high weight of competition. Despite the presence of unnecessary information in figure skating videos, human specialists can focus and reduce information when they evaluate jumps. In this study, we clarified the eye movements of figure skating judges and skaters while evaluating jumps and proposed a prediction model for jump performance that utilized specialists’ gaze location to reduce information. Kinematic features obtained from the tracking system were input into the model in addition to videos to improve accuracy. The results showed that skaters focused more on the face, whereas judges focused on the lower extremities. These gaze locations were applied to the model, which demonstrated the highest accuracy when utilizing both specialists’ gaze locations. The model outperformed human predictions and the baseline model (RMSE:0.775), suggesting a combination of human specialist knowledge and machine capabilities could yield higher accuracy.

## 1. Introduction

### 1.1. Background

Computer vision research tackles the task of not recognizing human actions in videos but evaluating the quality of the actions. Such tasks are referred to as action quality assessment (AQA) or human action evaluation (HAE) and cover sports skills, surgical procedures, and daily activities. Researchers are working on sports activity scoring tasks for sports skills to predict scores, like those of figure skating. Solving this task is expected to facilitate applications as a second opinion for judges and as a feedback tool for athlete performance [1]. AQA tasks have the following difficulties compared with typical action recognition tasks [2]:

- Because the samples are from the same action class, the differences among the instances are subtle;
- The entire action sequence must be “seen” to quantify the quality of action correctly and meaningfully;
- AQA datasets are very small compared to action recognition datasets.

In AQA task studies focusing on figure skating, some researchers have proposed a model to predict the entirety of technical elements, program components, and final total scores by using a 2 min 40 s ± 10 s short-program video as input to a video recognition model (such as C3D [3] and I3D [4]) [5,6,7,8,9]. Predicting the final score using the entire video as input may be a promising approach for predicting program component scores; however, predicting technical element scores is less useful for sports field applications. This is because of the change in the scoring rules for figure skating. Until 2004, the technical score was determined based on the entire performance of the program, evaluated on a relative scoring scale of 6.0 points. However, according to the current scoring criteria, scores are assigned to each technical element (the International Skating Union (ISU) judging system, in use since 2006) [10,11]. Thus, the current interest of skaters and coaches regarding technical element scores is not in the prediction of overall technical element scores from the entire performance video; rather, it is in the predicted scores of the individual technical element videos, such as one jump. The score for a single technical element is calculated based on the sum of the “base value”, which represents the difficulty of the element, and the “grade of execution” score (GOE), which represents the quality of the performed element. While the base value is judged based on the type of elements, the grade of execution score, evaluated on an 11-point scale from −5 to +5, leaves room for interpretation of the evaluation criteria, as shown in Table 1. Thus, there is an urgent need for prediction results based on mathematical models as a second opinion for skaters and coaches. In this study, we focus on jumps, which account for a large percentage of scores among technical elements, and tackle a new task: predicting the grade of execution score of a single jump.

In a previous study on the GOE in figure skating jumps, Hirosawa et al. proposed a new evaluation index using kinematic data from a tracking system [12]. In figure skating, the Ice Scorp tracking system was introduced at the 2019 World Championships for the first time at an official ISU-sponsored competition to visualize skaters’ performance quantitatively. This system can visualize vertical height, horizontal distance, and landing speed [13]. However, in a previous study, only numerical information obtained from the tracking data was used, implying that only features corresponding to certain aspects of the two evaluation criteria in Table 1 were used [12]. Therefore, prediction accuracy can be improved by adding video features to cover other evaluation criteria. In addition, based on the criteria of the GOE score, it is difficult to accurately determine where the judges focus their attention during the evaluation of jumps. Therefore, specialists may perform specialist-specific visual search behaviors during evaluation. In fact, judges must focus on the most relevant information during a competition and ignore irrelevant information. Research in sports psychology has examined visual search behaviors specific to professionals [14,15]. However, there are no studies on the visual behavior of figure skating professionals. If the information-selecting behavior unique to specialists during the evaluation of figure skating jumps can be clarified and the associated findings can be incorporated into a prediction model, the prediction accuracy is expected to improve. Thus, this study addresses the more field-oriented task of GOE scoring of figure skating jumps in AQA sports activity scoring. We attempted to improve the model using the following two approaches:

- Using kinematic data from a tracking system in addition to video features;
- Enabling the model to learn the gaze location of competition specialists such as judges and skaters when inputting video features.

Specific considerations for this study are as follows:

(1). To examine the differences in eye movements between judges and skaters when evaluating the GOE score of figure skating jumps (Part A);
(2). To propose a model for the grade of execution score of figure skating jumps using kinematic data from the tracking system and specialists’ gaze location (Part B).

By proposing a new AQA model that integrates the findings of kinesiology and psychology for figure skating jump performance, we expect further development in AQA research and industrial applications. Our contributions can be summarized as follows:

- We observed a significant difference in the gaze location percentages between judges and skaters, when adjusting for the skaters’ technical abilities. Both groups primarily focused on the upper body; however, the skaters tended to focus on the face, while judges focused more on the lower body and skating boots. These findings offer a new perspective for research on the eye movement patterns of sports officials;
- We proposed a prediction model that utilized kinematic data from tracking systems, specialist gaze locations, and image information. This model enhanced the accuracy of the baseline model, suggesting new possibilities for human–machine collaboration in AQA tasks.

### 1.2. Related Work

#### 1.2.1. AQA of Figure Skating

Action quality assessment (AQA) in figure skating has been a subject of interest for several researchers. A typical task is the prediction of short-program scores using machine learning and deep learning algorithms on video datasets. For instance, Pirsiavash et al. created a dataset, referred to as MIT-Skate, comprising 150 videos and proposed a model that predicted scores from pose features [5]. Similarly, Li et al. introduced a spatial–temporal pose extraction module and used skeletal information features [6]. In contrast, Parmar and Morris proposed a model that extracts RGB-based features using a three-dimensional convolutional neural network (C3D) instead of pose features [7]. Xu et al. created a new Fis-V dataset containing 500 videos. They proposed a multi-scale convolution model using long short-term memory (LSTM) to aggregate information from individual clips to accommodate the characteristics of figure skating competitions, which have longer performance times and more information with a lower impact on scores [8]. Han et al. propose a multi-scale location attention module (MS-LAM) to capture location information and multi-scale location-attentive long short-term memory (MLA-LSTM), which can efficiently learn local and global sequence information in each video [9]. However, these studies did not focus on predicting individual technical elements and relied solely on image features from the broadcast video for prediction.

Table 2 provides a comparison of existing AQA methods in figure skating.

#### 1.2.2. Performance Analysis of Figure Skating Jumps in Sports Sciences 

In sports science research, kinematics and kinetic parameters are commonly calculated using high-speed cameras and motion capture systems. However, only a few studies have been conducted on jumps during figure skating competitions. Sakurai et al. examined the differences in the average jump height for different jumps performed in a ladies’ singles competition at the 1998 Nagano Olympics [16]. King et al. compared the kinematic parameters of triple and quadruple toe-loop jumps performed in the men’s single category at the 2002 Salt Lake City Olympics [17]. After introducing the ISU judging system, Hirosawa et al. clarified the relationship between kinematic features and the grade of execution score of double-axel jumps in the ladies’ single short program at the 2019 World Championships using tracking technology for media contents [12].

#### 1.2.3. Eye Movements of Sports Judges Research in Sports Psychology

Sports officials must visually explore relevant information to make accurate decisions [18]. Specifically, they focus on the most relevant information during competition and ignore irrelevant information [14]. Sports officials can be classified into three categories: interactors, reactors, and monitors, based on the amount of interaction with the players, degree of physical activity required, and number of cues [19]. By this definition, figure skating judges are classified as monitors based on their minimal interaction with athletes, minimal demand for physical activity, and high number of cues. However, no studies have been conducted on the eye movements of figure skating judges. Regarding the relationship between judging accuracy and internal factors, athlete experience has been found to be the most critical of the three internal factors (judging experience, athlete experience, and spectator (viewing) experience) in trampoline judging [20]. In gymnastics balance beam judging, it has been reported that those who can perform the judged techniques exhibit a higher ability to detect errors in joint angles [21]. Furthermore, different specialists (motor specialists/visual specialists/novices) have different kinematic characteristics. However, the visual specialist group includes referees and coaches [22]. Thus, these previous studies suggested that the accuracy of technical evaluation and the cues considered necessary during evaluation differ depending on the internal factors of the evaluators. In the case of monitors, it has been reported that the influence of athlete experience is significant, primarily on whether the judges themselves can perform the technique to be judged. In addition, studies on the eye movements of monitors have reported mixed results because of variations in experimental conditions and variable controls. 

One primary consideration is the difference between highly experienced and less experienced judges. When comparing more and less experienced judges, more experienced judges tended to have fewer fixations, but the difference was not significant [23]. In the gymnastics vault, the judgment accuracy was higher at the higher license level, indicating that the body parts to be gazed at differed between the higher and lower license levels. In addition, a comparison of judges who could and could not perform the technique showed that judges who could perform the technique had a number of fixations on the athlete, and the two groups had different focus points. However, in contrast to [20], judges who could not perform the technique were less accurate in their judgments [24]. Research on rhythmic gymnastics suggests that, unlike national judges, international judges make technical judgments without relying on visual fixation [25]. 

Another consideration is the comparison of eye movements among players, coaches, and judges. Eye movements have been found to differ depending on the roles of the judge, coach, and player, with players showing significantly greater fixation duration near the hips and lower extremities. The accuracy of judgment was the highest in the order of judges, coaches, and players; however, there was no significant difference, and this study was a case study of one subject at a time [26]. In gymnastics floor events, the accuracy of judgments was highest in the order of motor specialists (athletes), novices, and visual specialists (coaches and judges). However, there were no significant differences in fixation time or number of fixations among the three types of specialties. Notably, the visual specialist group included both referees and coaches [27]. 

Although a number of studies on monitors are listed above, it has been reported that most sports officials’ studies have focused on interactors, and there are few studies on reactors and monitors [28]. In addition, the aforementioned studies’ designs were not based on the theory of visual search behavior or specialty. The experimental design must consider ecological validity. Moreover, the study objectives and explanatory variables were diverse and varied from study to study, and whether the study results from a specific sport can be applied to other sports has not been examined [14].

## 2. Methods

As this study tackled two issues, the methods are described separately for each, and this study was conducted in accordance with the Declaration of Helsinki and approved by the Ethics Committee of Keio University (protocol codes 2021-96 and 2021-08-23).

### 2.1. Method of Specialists’ Eye Movements—Part A

Before developing the AQA model, we analyzed the differences in eye movements between judges and skaters while assessing the GOE scores of figure skating jumps. Eye movement research in sports suggests a tradeoff between internal and external validity in laboratory and field experimental environments, and researchers should aim to optimize both [28]. This study improved internal validity by conducting a laboratory experiment instead of a field experiment in a figure skating competition.

#### 2.1.1. Participants

This study involved a judge group (three subjects: one male and two females) and a skater group (three subjects: three females). The selection criteria for each group were as follows:

1. Judges: Subjects held a judge’s qualification approved by the National Federation and were not registered as skaters with the federation in the year the experiment was conducted. They also had passed a grade 6 technical test for skaters during their competitive skating career;
2. Skaters: Subjects were registered with the National Federation, and they had passed a grade 6 technical test for skaters.

The Japan Skating Federation (JSF) certifies judging qualifications, which are categorized into five levels (NR, N, A, B, and T, from high to low). All three subjects in the judge group held a B grade. The JSF also sets nine test grades for competitive skaters (8, 7…1, and beginner grade, from high to low) to represent their competitive technical abilities. This study focused on the double-axel jump, included in the grade 6 technical test. Hence, the subjects had the same athletic ability and specific motor experience that they judged in this study.

#### 2.1.2. Task

Subjects watched 30 jump videos and assigned GOE scores for each jump on an 11-point scale from −5 to +5 (including 0), as performed in competition [10,11].

#### 2.1.3. Video

The video featured 30 double-axel jumps from the ladies’ short program at the 2019 World Championships, each of which received a GOE score of 0 or higher from all judges. Figure skating jumps last only a few seconds. However, in actual competitions, judges evaluate jumps within a sequence of performances and movements. Thus, each jump video in this experiment was approximately 30 s long, including the performances before and after the jump. The video, created from the ISU’s official YouTube channel data [29] at an FPS of 29.97, included program music and audience cheers but no commentary. We obscured the provisional scores during the performance and created two videos (Video 1, Video 2), each containing 15 first-half jumps and 15 s-half jumps. The order of jumps followed that of the actual competition.

#### 2.1.4. Eye Tracker

We used a Tobii Pro Spectrum (Tobii AB, Stockholm, Sweden) to track the subject’s gaze (both eyes). The display size was 23.8 inches, and the resolution was 1920 × 1080. The fixation filter settings, recommended by [24,30], were set to a minimum fixation duration of 60 ms and a measurement frequency of 60 Hz.

#### 2.1.5. Procedure

We provided the following information and instructions to the subjects in advance:

- All jumps are 30 double-axel jumps in the ladies’ senior category and had no under-rotation or other technical deductions;
- GOE guidelines (same as Table 1);
- Subjects must wait until the response time appears on the screen, observe the video, and move their eyes as naturally as possible without moving their face.

The subjects evaluated three jumps for practice and were informed that these jumps had a GOE of zero, which served as a basis for judging this experiment. The practice jumps, performed in competition, were excluded from the video because some judges gave negative scores, even though the final GOE scores were close to zero. The actual GOE scores were 0.05, 0.05, and −0.05.

#### 2.1.6. Eye Tracking Experiment

**Figure 1:**
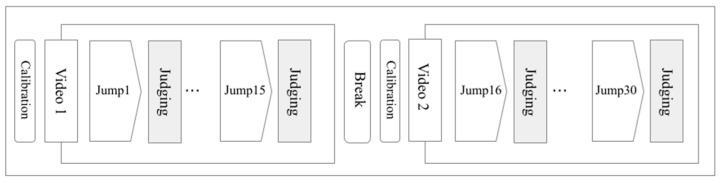
Experiment flow of tracking specialists’ eye movements. Subjects watched Videos 1 and 2, each containing 15 jumps, and scored each jump. There was a break between Videos 1 and 2, and subjects calibrated their gaze before watching each video.

The experimental flowchart of this study is shown in Figure 1. Subjects first evaluated jumps using Video 1, with each jump evaluation lasting 15 s. After evaluating the 15 jumps in Video 1, subjects took a 10 min break. They then evaluated the 15 jumps shown in Video 2. Subjects underwent gaze calibration before viewing Videos 1 and 2, and each jump was preceded by a 5 s pause in the video.

#### 2.1.7. Variables

**Figure 2:**
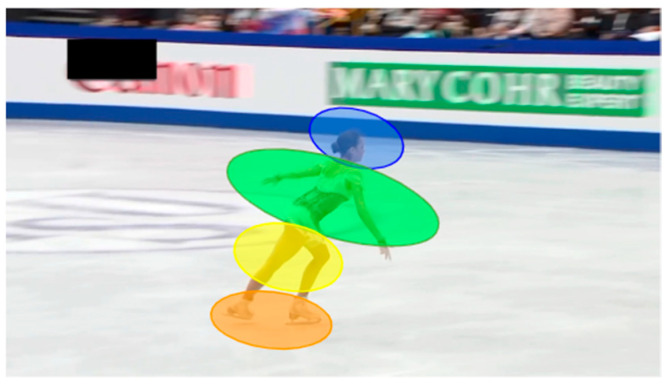
Definition of areas of interest (AOI). We categorized the proportions of gaze placement into four AOI: (1) face, (2) upper body, (3) lower body, and (4) boots.

In addition to the measurement variables based on the information reduction hypothesis, as reported in [31], we calculated the percentage of gaze locations based on frame-by-frame analysis, as reported in [32,33]. The variables can be summarized as follows:

- Judgmental performance
  Experience as an athlete, particularly the ability to perform a technique to be judged, influences judgmental performance [20]. In this study, we expected no group differences in judgment errors between the judge and skater groups because there was no difference in their competitive ability as skaters. As in previous studies on sports judges research in sports psychology, the mean absolute error (MAE) was used as the evaluation index in this study;
- Number of fixations
  Based on the information reduction hypothesis, specialists pay more attention to the task-relevant parts [31]. As judges have a specific specialty in evaluating the performance of figure skating jumps, the judge group is expected to have a longer fixation duration on the parts of the jumps related to the task;
- Fixation duration
  Based on the information reduction hypothesis, specialists have more time to pay attention to the task-relevant parts of the task [31]. As judges have a specific specialty in evaluating the performance of figure skating jumps, the judge group is expected to have a longer fixation duration on the parts of the jumps related to the task;
- Gaze location
  Based on the two-dimensional coordinates obtained by the eye tracker, we calculated the number of frames and the percentage of gaze locations for the subjects using frame-by-frame analysis [32,33]. The area of interest (AOI) based on the body parts was defined into four areas, as shown in Figure 2. We hypothesized that there would be a difference in percentage between the professionally educated judges’ group and the skaters’ group, with specialists placing their eyes on more task-relevant areas.

#### 2.1.8. Analysis

As mentioned previously, a single video of one jump used in this experiment was approximately 30 s long, including footage before and after the jump. However, we only used the part corresponding to the jump for data analysis. Therefore, the following procedure determined the frames to be analyzed:

1. A researcher in this study, who had experience in figure skating, determined the take-off point for each jump based on the video frame;
2. The starting point of the analysis was 150 frames (approximately 5 s) before the take-off point because the criteria included the execution of steps and unexpected or creative entries before the jump in evaluating the GOE score;
3. Eighty-nine frames (approximately 3 s) after the take-off point were set as endpoints for analysis. Based on the above definition, 240 video frames (29.97 FPS) were selected for the analysis;
4. As defined above, 480 frames (60 Hz) of eye-tracking data acquired by the eye tracker were used for analysis.

We conducted a repeated-measures two-way ANOVA with two factors: difference in subjects’ attributes (judges and skaters) and differences in the videos (Videos 1 and 2). This was performed to evaluate judgment performance, the number of fixations, and the duration of fixations. The difference in attributes was considered a fixed effect, while the difference in videos was considered as a variable effect. If the analysis of variance proved significant, a multiple comparisons test (Tukey’s HSD) was performed on all combinations as a post-test. Additionally, we conducted a χ-square test on a 2 × 4 contingency table to determine the percentage of gaze locations for different attributes (judges/skaters) and AOI (face/upper body/lower body/boots). The significance level for rejection was set to 5%. JMP16 was used for statistical analysis.

### 2.2. Method of Predictive Model of Figure Skating Jump—Part B

#### 2.2.1. Dataset

We created the following four datasets:

- Video
  As in Part A, we used 30 videos of double-axel jumps rated 0 or higher in GOE by all judges in the 2019 World Championships ladies’ short program. Each jump video contained 240 frames, and provisional scores were blacked out;
- Kinematic features
  Ice Scorp, a tracking technology for media content used to visualize skater jump data, was implemented in the competition. For detailed information on Ice Scorp, please refer to previous papers [12,13]. The vertical height, horizontal distance, and speed after landing on ice (landing speed) were obtained for each of the 30 jumps. We were provided these data from Qoncept Inc., which is developing a tracking system;
- Gaze location
  Two-dimensional eye coordinates (480 frames) of the six specialists (three judges and three skaters) for each jump video were obtained in the Part A experiment;
- Ground truth (GT) of GOE score
  We extracted the GOE score for each jump provided by each judge from the official results page of the competition [34]. Note that the GOE score used in this study was the trimmed average of nine judges and incorporated values in the range of 0–5.

#### 2.2.2. Proposed Model

**Figure 3:**
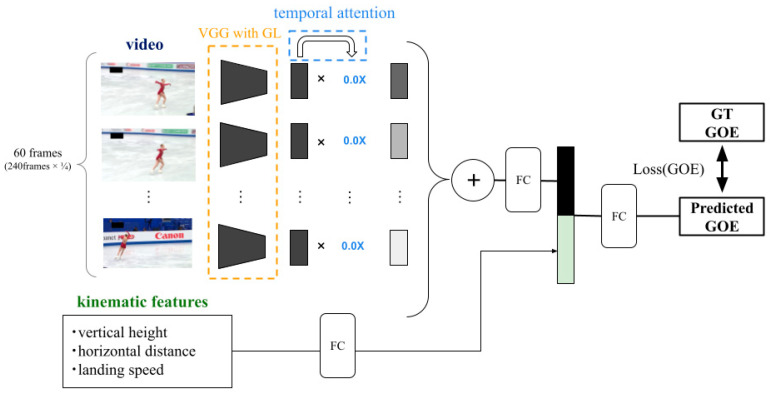
Overview of our proposed model. The video is used as input and information reduction based on the gaze location of a human specialist is achieved by passing it through VGG with gaze location (GL). Then, temporal attention is multiplied to obtain features from the video. Finally, the kinematic features are added, and the GOE is predicted.

**Figure 4:**
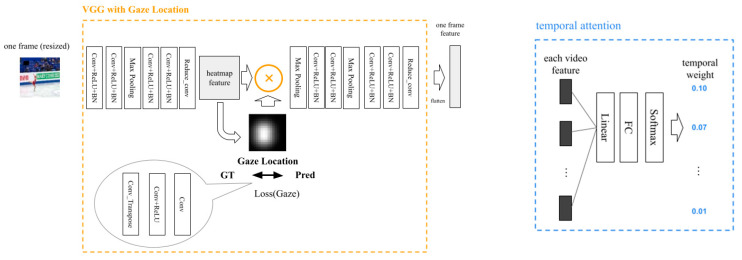
The detailed structure of VGG with gaze location and temporal attention. The most unique point of this model is the reduction in redundant information by utilizing specialists’ gaze location for RGB-based video features. In addition, by using temporal attention, the model considers the time-series nature of video information.

We proposed a predictive model for the performance of figure skating jumps that utilizes kinematic data and specialists’ gaze location in addition to videos (Figure 3). Our proposed model used kinematic data obtained from the tracking data as input features. However, kinematic data can satisfy only two of the six evaluation criteria for GOE scoring [12]. By adding video features to the proposed model, we can extract relevant features for other GOE criteria. The main contribution of the proposed model isa VGG with gaze location (Figure 4). As the name suggests, the model structure of the VGG with gaze location is based on the VGG16 model [35]. This study focused on a single category of figure skating competition, and there was only one competitor on the ice.

However, the videos had redundant information regarding assessing the jump quality. To further narrow the focus, we utilized specialists’ gaze information in the videos. A gaze heat map (GT GL) was created using a Gaussian kernel for the specialists’ eye coordinate data measured in Part A, and a predicted gaze map (pred gaze) was obtained for the intermediate features extracted by the convolution layer. By multiplying the features by the pred gaze, the image features were masked, and the focus points were narrowed down, which was expected to improve the prediction accuracy. Subsequently, the model multiplied the temporal attention to obtain the final image-based features. Finally, the kinematic features obtained from the tracking system and image features were combined to predict the GOE score.

#### 2.2.3. Accuracy Verification Experiment

- Points of consideration

**Figure 5:**
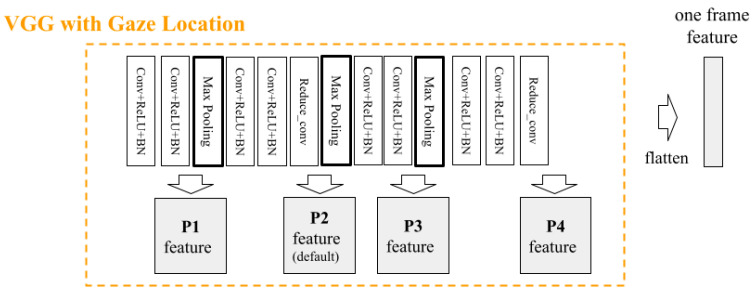
Where to place and multiply specialists’ gaze location. We examined places where video feature information reduction by specialists’ gaze location should be implemented in the network. P1–P4 were set as the shallowest to the deepest layer. P2 was designated as the default position.

We examined the following two items to demonstrate the effectiveness of the proposed model:

1. Utilizing specialists’ gaze location: We compared the proposed model with a baseline model to demonstrate its effectiveness and examined which of the specialists’ gazes should be used in the proposed model (three skaters, three judges, or all six);
2. Where to place specialists’ gaze location in the network: We examined where it would be most effective to implement information reduction using the specialist gaze in the network. As shown in Figure 5, we set P1–P4 as the shallowest to the deepest layer and examined how the accuracy changed when the gaze was multiplied by each of them. For comparison with the baseline model, all the models were implemented in P2 as the default position, which was the middle layer.

- Implementation details

The dataset was trained with 30 epochs using Adam as the optimization algorithm, a learning rate of 0.001, and a batch size of 2. The loss function (Loss) was calculated by weighting loss (GOE) and loss (Gaze) and using the coefficients (a = 1, b = 0.01) added together. Loss (GOE) was defined as the root mean squared error between the predicted value of GOE (Pred) and GT. Loss (Gaze) was used for focal loss, as described previously [36]. For the ground truth of the key point, the specialists’ gaze coordinates, a heat map was created using a Gaussian kernel, as in [37]. This heat map was designated as GT (Gaze). The size of the object was set to 2.0. The hyperparameters α and β of focal loss were set to 2.0 and 4.0, respectively. N is the number of gaze coordinates (key points) of the specialists used (only judges or skaters: N = 3; all subjects: N = 6).

$$Loss\;(\mathrm{Gaze})=\frac{-1}{N}\sum_{xyc}\;\left\{\begin{array}{ll} {\left(1-{Pred}_{xyc}\right)}^{\alpha }\log\left({\mathrm{Pred}}_{xyc}\right) & if\;GT_{xyc}=1\\ {\left(1-GT_{xyc}\right)}^{\beta }{\left({\mathrm{Pred}}_{xyc}\right)}^{\alpha }\log\left(1-{Pred}_{xyc}\right) & otherwise \end{array}\right.$$

- Evaluation

The leave-one-out method was used to evaluate the models because the dataset was small (30 samples). Specifically, the model was trained on 29 data points and tested on 1 data point, and its accuracy was determined by considering an average of 30 iterations. The evaluation metrics were the root mean squared error (RMSE) and Spearman’s rank correlation coefficient (Corr.) between the predicted and GT values. Although prior research in sports psychology has used MAE and, in the field of AQA, has used rank correlation, we believe that, in the field of sports competitions, large prediction errors should be minimized to the best extent possible. Therefore, we emphasized the RMSE results in our integrative discussion.

## 3. Results

### 3.1. Result of Specialists’ Eye Movements—Part A

#### 3.1.1. Judgment Performance

Table 3 summarizes the MAE between the GT scores of the judges in the actual competition and those of the subjects in the experimental environment. An error of approximately 1.0 was observed in both groups. In the present study, the absolute error was smaller in the skater group than in the judge group, and the within-group variance was also smaller. Repeated-measures two-way ANOVA revealed significant differences only for the video factor (F = 9.835, *p* = 0.002, partial η^2^ = 0.050) (Table 4), with poor accuracy in determining Video 2 in both groups. The attribute factors (F = 4.450, *p* = 0.103, partial η^2^ = 0.011) and their interactions did not differ significantly (F = 2.519, *p* = 0.114, partial η^2^ = 0.013). Therefore, we could not reject the null hypothesis that judges and skaters have no difference in judgment accuracy.

#### 3.1.2. Number of Fixations

Table 5 summarizes the results for the number of fixations. Repeated-measures two-way ANOVA revealed no significant differences in the attribute factor (F = 0.059, *p* = 0.820, partial η^2^ = 0.004), video factor (F = 0.134, *p* = 0.715, partial η^2^ = 0.001), or interaction (F = 1.936, *p* = 0. 166, partial η^2^ = 0.050). Therefore, we could not reject the null hypothesis that there is no difference in the number of fixations between judges and skaters. Fixation occurs when the eyes are fixed on a visual target, perception is stable, and the eyes receive visual information [38]. Based on the information reduction theory [31], we expected judges to have more fixations to efficiently search for information relevant to the decision, even in the complex and fast movements of skaters, because of their specialty in this task. However, the results of this study indicated no difference in the number of fixations between the two groups. In previous studies on the effect of fixation numbers on differences in specialty, certain studies reported the athletes having more fixation numbers [26] and no difference in the subjects’ attributes [27]. In comparisons among judges, Pizzera et al. reported that the number of fixations differed depending on whether they had specific motor experience in performing the skill they were judging [24]. The results of this study provide a new perspective in that there is no difference in the number of fixations between the judge and skater groups when controlling for technical ability among skaters, which may be a confounding factor.

#### 3.1.3. Fixation Duration

Table 6 and Table 7 summarize the fixation duration during video viewing. Repeated-measures two-way ANOVA revealed no significant differences in the main effects of the attribute factor (F =2.493, *p* = 0.190, partial η^2^ = 0.068) or video factor (F =1.113, *p* = 0.293, partial η^2^ = 0.005). However, the interaction differed significantly (F = 11.767, *p* = 0.001, partial η^2^ = 0.050). Tukey’s HSD test results exhibited a statistically significant difference between Judges Video 2–Skaters Video 2 (*p* = 0.037, Cohen’s d = 0.945) and Skaters Video 1–Skaters Video 2 (*p* = 0.010, Cohen’s d = 0.625). Therefore, the null hypothesis that there is no difference in fixation duration between judges and skaters was rejected for the second half of the video viewing, as the duration the judge group spent fixated on the second half of the video was significantly longer than that of the skaters. Based on information reduction theory [31], we expected that judges would have a certain level of specialty in evaluating the performance of figure skating jumps and, therefore, the judge group would have a longer fixation duration. Prior studies have indicated mixed results, with certain case studies reporting a longer fixation duration for skaters [26] and others reporting no differences by subject attributes [27]. In addition, Pizzera et al. reported no difference in the fixation duration between judges with and without specific motor experience [24]. In this study, the judge group exhibited a significantly longer fixation duration than the skater group, but only for the latter half of the video. This result differs from those of previous studies.

#### 3.1.4. Gaze Location

Table 8 presents the results of a contingency table that tabulates the percentage of gaze location for each group using frame-by-frame analysis [33,34] for all jumps; missing values owing to blinking were excluded. Both the judge and skater groups looked at the upper body more frequently than the lower body. However, the judge group looked at the face less frequently and looked at the lower body and boots more frequently. A χ-square test was performed on the proportions between groups, and the *p*-value was significant at the 1% level (χ^2^ (3) = 2653.828, *p* < 0.001, Cramer’s V = 0.190). Therefore, the null hypothesis that the judge and skater groups have the same percentage of gaze locations was rejected. Precise frame-by-frame gaze location was not considered in previous studies that focused on monitors. This study suggested that the percentages of gaze locations differed between the two groups. Specifically, while both groups focused on the skater’s upper body, the skater group had a higher proportion of focus on the faces than the judge group, which focused on the lower body and boots. Moreover, the judge group showed a decrease in the percentage of boots and an increase in the proportion of faces in Video 2. The percentage of the upper body and face increased, and that of the lower body and boots decreased in the skaters’ group (Table 9).

### 3.2. Result of Predictive Model of Figure Skating Jump—Part B

Table 10 presents the accuracy results of each model. As a baseline model, the model combining both kinematic features and video (RGB image features) yielded the best accuracy in all evaluation metrics (0.954 for RMSE and 0.338 for rank correlation coefficient) compared to only kinematic features or only video features. Our proposed model using all six subject gaze locations and applying the temporal attention module was the most accurate for both RMSE and rank correlation coefficient, with an RMSE of 0.775 and a rank correlation coefficient of 0.697.

**Figure 6:**
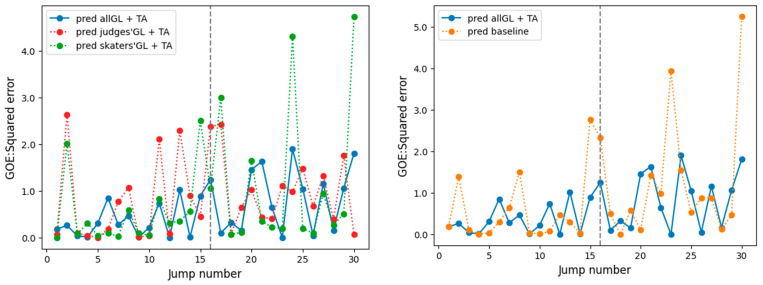
Comparison of ground truth and each model’s predicted score of GOE. Note: the jump numbers are based on the competition order, as in the Part A experiment. The gray line in the center indicates the boundary between Video 1 and Video 2. The predictions shown in blue are based on the final proposed model using all subjects’ gaze locations (Model No. 9); those in red are based on the judges’ gaze locations (Model No. 7); those in green are based on the skaters’ gaze locations (Model No. 8); and those in orange are those of the baseline model (Model No. 3).

Figure 6 shows the square error comparison for the model combining all six subjects’ gaze locations and temporal attention (predicted all GL + TA, No. 9, our proposed model), the model combining judges’ gaze locations and temporal attention (predicted skaters’ GL + TA, No. 7), the model utilizing skaters’ gaze locations (predicted judges’ GL + TA, No. 8), and the baseline model (no gaze location, No. 3). Although the skaters and judges models each exhibited significant errors in their predictions, the errors were generally minor in the proposed model, which combined the gaze locations of the two groups. In particular, Figure 6 shows that the judges model (red line) had a jump in Video 1 (jump number 1–15) and the skaters model (green line) had a jump in Video 2 (jump number 16–30), where the square error was larger. However, the proposed model had a smaller error for each.

We tested for change in accuracy by changing the position in which the gaze location is multiplied by the features obtained from the convolutional layers (Figure 5). Table 11 indicates that the best accuracy was obtained when multiplying the gaze location at P2, and the accuracy decreased for all evaluation indicators at other locations. Based on the above results, the final model proposed in this study used the gaze locations of all six subjects and multiplied them at the center position (P2) of the convolution layer.

Finally, Table 12 shows an integrated comparison of the prediction accuracy of the human judge and skater groups introduced in Part A with the model proposed in our study. As mentioned above, RMSE was used for the integrated comparison because it is a better model for reducing large errors with GT. Consequently, the accuracy was better than that of the judge and the skater groups.

## 4. Discussion

For judgment performance, we determined a judgment error (MAE) of approximately 1.0 for both the judge and skater groups. The results of the repeated-measures two-way ANOVA indicated no significant differences in the main effect of the attribute factor. This implied that, on average, there was no difference in judgment accuracy between the two groups. This study validated judgment performance under the condition that all subjects had the same technical test grade and specific motor experience as those related to the double-axel jump. Therefore, the results partially support previous studies showing that athletes’ specific motor experiences influence the judgment of monitors [20,21]. As in the case of gymnastics, evaluating the GOE score of figure skating jumps also suggests that the judges use their own experiences as skaters to make decisions. The primary effect of the video factor was significant, and the judgment error was more prominent for Video 2 in both groups. As mentioned earlier, the order of the jumps used in this study was based on that of actual competitions, with Video 2 featuring the top-ranked skaters in the world. A previous study reported reputational bias in the technical judgment of figure skating based on whether the skater is known to the competitor [39]. Therefore, the participants in this study were also influenced by a reputation bias when identifying athletes, particularly in Video 2. Although, in judgment performance, the interaction between the attribute factor and the video factor was not significant, the 95% confidence interval (CI) of the MAE indicated that the absolute error for the judge group in Video 2 was large. A detailed examination of the gaze locations of the two groups revealed a significant difference between the results of skaters and judges. While both groups focused on the upper body, the skaters focused on the face, whereas the judges focused on the lower body. The judge group also paid more attention to the boots than the skater group. This suggests that the judges place their gaze on areas more directly related to the evaluation of technical aspects, such as the steps and footwork before jumping, blade movement during stepping, take-off, and landing, and the tightness of the axis of rotation of the lower limbs in the air. When comparing gaze locations between Videos 1 and 2, the percentage of skaters’ gaze locations on the lower body and boots decreased in Video 2, while placement on the face and upper body increased. However, in Video 2, the judge group had a decreased fixation on boots and an increased fixation on faces, which was closer to the gaze location in the skater group. In addition, the interaction between attributes and video factors was significant for fixation duration, with the judge group having significantly longer fixation times in Video 2. The above results suggest that judges may be searching for information necessary for the evaluation of the lower limbs and boots while focusing on the upper body. In Video 2, where there were more famous, high-ranking skaters, the percentage of gaze locations being designated to boots decreased and that being designated to faces increased, and the fixation duration was longer than in Video 1, which can be assumed to have caused a decrease in judgment accuracy. We inferred that the subjects in this study were not top-level judges. They judged with a different gaze location from the skater group while still using their experience as skaters. However, the participants were still learning eye movements when judging, and it is thought that the different gaze locations in the second half of the video compared to those in the first half led to a decrease in the accuracy of their judgments. Judges with the same skill level as the skaters and a higher level of judging qualification would be expected to make more accurate judgments, and their eye movements would be expected to be similar to those of the judges in Video 1 of the subjects in this study. While controlling for technical test grades as a skater and specific motor experience, research cooperation with subjects with even higher-level judging qualifications is necessary; however, this will be an essential issue to consider in the future. There was no significant difference in the number of fixations between the two groups. Results of previous studies on the effect of different specialties on the number of fixations were mixed, with [26] indicating a higher number of fixations for players and [27] exhibiting no difference according to subject attributes. When comparing judges, [24] reported that the number of fixations differed depending on whether they had specific motor experience. In the context of these discussions, the results of this study provide a new perspective in that there is no difference in the number of fixations between the judge and skater groups after controlling for the technical ability of skaters, which may be a confounding factor. Our results support the idea that specific motor experience is related to the number of fixations of the judges [24] and that the number of fixations does not vary with the subject’s attributes [27].

**Figure 7:**
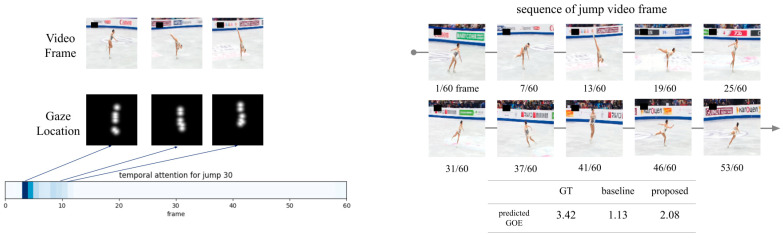
Case study of jump number 30 (baseline model vs. proposed model). Note: An example of improved accuracy in the proposed model compared to the baseline model. The darker the color of the temporal attention heat map, the greater the weight for predicting GOE. Temporal attention focuses on the skater’s unique posture before the take-off, which is consistent with GOE evaluation criterion 4 shown in Table 1. Prediction accuracy is improved by considering aspects that are not considered from the kinematic features or averaged video features without considering the temporal attention.

In the GOE prediction accuracy of the AQA model, which utilized the gaze location of specialists, the combination of differences in gaze location between judges and skaters resulted in improved accuracy. When only training the gaze locations of each group, the accuracy was higher when utilizing those of the skaters, similar to the experimental results of subjects in Part A; however, even better accuracy was achieved by combining the gaze locations of the two groups. Temporal attention also showed a positive effect when both sets of gaze locations were combined. The importance of temporal information appears to differ when evaluating jumps. By combining the gaze locations of judges and skaters with only the information considered necessary in the time direction, it can be inferred that the AQA model was constructed to acquire information more directly related to technical evaluation, which is challenging to collect through skater experience alone. Figure 7 shows the output results of jump number 30 as an example of the significant improvement in the accuracy of the proposed model compared with the baseline. From the temporal attention heatmap, frames 4–12 were highly important for predicting the GOE. The jump video shows the skater skating backward, with the right foot holding a high (spiral position) during these frames. This behavior would fit into criterion 4, “steps before the jump, unexpected or creative entry”, of the guidelines listed in Table 1. Such localized characteristics related to jump quality in the temporal direction cannot be obtained from averaged video features without temporal attention. Similarly, kinematic features cannot be expressed. The proposed model can consider such local features along the temporal direction as well as the importance of the spatial direction with experts’ gaze location, leading to improved prediction accuracy.

As shown in Table 11, the prediction accuracy of the proposed model was better than that of both groups of human judges and skaters in the Part A experiment, suggesting that better accuracy than humans can be achieved in the difficult task of AQA by utilizing the combined knowledge of humans and machines.

Despite these contributions, this study has several limitations. One limitation of this study is the small size of the dataset. The tracking system has been implemented in only a few international figure skating competitions. This renders it difficult to obtain quantitative kinematic data related to a judge’s evaluations. The number of participants for whom gazes were obtained was also small, with three participants in each group. As summarized in Section 1, there are no reported studies of judges’ eye movement in figure skating. A larger dataset is essential for the progression of related studies, and collaboration between international/national federations and academic institutes/researchers is required to achieve this. In addition, while controlling for technical test grades as a skater and specific motor experience, which all subjects have, research cooperation with subjects with even higher judging qualifications is necessary. Another limitation is that this model does not consider harmony with music that is specific to figure skating AQAs. The need for a multimodal approach is asserted in AQA tasks. In particular, figure skating is highly related to music. The jump GOE criteria also include items related to music (Table 1). If the relationship between music and movement is successfully used as a feature, a more accurate model can be constructed. Finally, from the viewpoint of practical application, it is essential to aim not only for prediction accuracy but also for reduced computational cost. Although our proposed method reduces the input information, the computational cost increases because of the prediction of gaze location as an input feature. It will be essential to reduce the computational cost of AQA tasks.

However, despite these limitations, this study proposes a distinctive method that suggests new possibilities for human–machine cooperation in AQA tasks.

## 5. Conclusions

This study tackled a new AQA task in predicting the GOE score for figure skating jumps, which is more complicated than a general action recognition task. We proposed an estimation model with improved prediction accuracy by adding new kinematic features obtained from tracking data and reducing information by learning specialists’ gaze locations. First, we clarified the difference between skaters’ and judges’ visual behaviors. The judges behaved more under the information reduction hypothesis in terms of fixation time in the latter half of the video (Video 2). In addition, the ratio of gaze locations differed between the two groups. Although there was no difference between the two groups in terms of the fixation number on a specific AOI, a detailed frame-by-frame examination suggested that focus points may differ. However, the extent to which this difference in visual search behavior affects judgment remains unclear. The judges in this study were at a national level and cannot be considered to be at the top level of judgment. Therefore, it can be inferred that they are in the process of acquiring visual search skills as judges while utilizing their motor experience as skaters for evaluation. Then, we created a model that utilizes the gazes of both groups. The most accurate estimation model is one that learned the gaze location of all the subjects and added kinematic features. The results also indicated that information reduction using gaze locations should be multiplied at the center of the network. This study suggests new possibilities for human–machine cooperation in AQA tasks, and future developments in this field are expected.

## Figures and Tables

**Table 1:** Guidelines for marking the GOE score of a jump, positive aspect.

No.	Criterion
1	very good height and very good length (of all jumps in a combo or sequence)
2	good take-off and landing
3	effortless throughout (including rhythm in jump combination)
4	steps before the jump, unexpected or creative entry
5	very good body position from take-off to landing
6	element matches the music

Note: No. 1, 2 and 3 are mandatory for acquiring +4 and +5 [11].

**Table 2:** Comparison of exiting AQA in figure skating.

	Dataset	Data Type	Methods
[5]	MIT-Skate	Video (Pose)	Pose + DCT
[6]	MIT-Skate/Fis-V	Video (Pose)	STPE-ATRE-SP
[7]	MIT-Skate	Video (RGB)	C3D-SVR
[8]	MIT-Skate/Fis-V	Video (RGB)	SA-LSTM, MCS-LSTM
[9]	MIT-Skate/Fis-V	Video (RGB)	MS-LAM, MLA-LSTM

**Table 3:** Judgment performance per subject and between groups.

Subject	MAE ± SD	95% CI
Judge A	1.181 ± 0.662	0.934–1.428
Judge B	0.986 ± 0.786	0.692–1.279
Judge C	1.010 ± 0.679	0.756–1.263
Skater A	0.871 ± 0.595	0.649–1.094
Skater B	0.900 ± 0.668	0.650–1.150
Skater C	0.971 ± 0.627	0.737–1.206
Judges	1.059 ± 0.107	0.794–1.323
Skaters	0.914 ± 0.052	0.786–1.042

Note: MAE = mean absolute error; SD = standard deviation; CI = confidence interval.

**Table 4:** Judgment performance per group per video.

Group	Video	MAE ± SD	95% CI
Judges	1	0.825 ± 0.611	0.646–1.004
	2	1.292 ± 0.751	1.073–1.511
Skaters	1	0.837 ± 0.593	0.664–1.010
	2	0.991 ± 0.674	0.794–1.188

**Table 5:** Number of fixations per subject and between groups.

Subject	Number of Fixations ± SD	95% CI
Judge A	11.167 ± 2.705	10.157–12.177
Judge B	13.800 ± 3.872	12.354–15.246
Judge C	16.467 ± 2.837	15.407–17.526
Skater A	15.533 ± 2.801	14.488–16.579
Skater B	12.167 ± 3.130	10.998–13.336
Skater C	12.367 ± 2.822	11.313–13.420
Judges	13.811 ± 1.536	9.994–17.628
Skaters	13.356 ± 1.707	9.114–17.597

**Table 6:** Fixation duration per subject and between groups.

Subject	Fixation Duration (ms) ± SD	95% CI
Judge A	5465.833 ± 1098.456	5055.663–5876.004
Judge B	6446.333 ± 1037.293	6059.002–6833.665
Judge C	6305.867 ± 859.441	5984.946–6626.787
Skater A	5247.500 ± 1183.477	4805.582–5689.418
Skater B	5173.033 ± 908.389	4833.835–5512.231
Skater C	5941.400 ± 1169.816	5504.584–6378.216
Judges	6072.678 ± 188.735	5603.833–6541.523
Skaters	5453.978 ± 145.550	5092.412–5815.543

Note: ms = milliseconds.

**Table 7:** Fixation duration by each group for each video.

Group	Video	Fixation Duration (ms) ± SD	95% CI
Judges	1	5892.311 ± 1062.856	5581.772–6202.850
	2	6253.044 ± 1085.546	5935.875–6570.213
Skaters	1	5794.511 ± 804.047	5559.589–6029.433
	2	5113.444 ± 1315.342	4729.135–5497.753

**Table 8:** Comparison of gaze location between groups based on AOI.

Overall				
(Frame)	Face	Upper Body	Lower Body	Boots
Judges	8857	17,461	9183	2560
Skaters	13,130	15,946	5333	909
(%)				
Judges	23.27%	45.88%	24.13%	6.73%
Skaters	37.18%	45.15%	15.10%	2.57%

Note: AOI = area of interest; see Figure 2 for a detailed definition of AOI.

**Table 9:** Differences in gaze location between groups based on AOI depending on the video.

Video 1				
(Frame)	Face	Upper Body	Lower Body	Boots
Judges	3809	8807	4468	1790
Skaters	6081	8291	2733	528
(%)				
Judges	20.18%	46.66%	23.67%	9.48%
Skaters	34.48%	47.01%	15.51%	2.99%
Video 2				
(Frame)	Face	Upper Body	Lower Body	Boots
Judges	5048	8654	4715	770
Skaters	6285	8069	1925	140
(%)				
Judges	26.31%	45.10%	24.57%	4.01%
Skaters	38.28%	49.14%	11.72%	0.85%

**Table 10:** Accuracy comparison between the proposed and baseline models.

No.	Model	RMSE	Corr.	MAE
1	KF	1.200	0.020	0.978
2	Video	1.141	0.184	0.890
3	KF + Video (baseline model)	0.954	0.338	0.767
4	KF + Video + judges’ GL	0.900	0.509	**0.632**
5	KF + Video + skaters’ GL	0.816	0.655	0.741
6	KF + Video + all GL	0.908	0.544	0.741
7	KF + Video + judges’ GL + TA	0.930	0.431	0.735
8	KF + Video + skaters’ GL + TA	0.924	0.516	0.797
9	**KF + Video + all GL + TA**	**0.775**	**0.697**	0.653

Note: RMSE = root mean square error; Corr. = Spearman’s rank correlation; KF = kinematic feature; GL = gaze location; TA = temporal attention. The bold and underlined values are the most accurate.

**Table 11:** Accuracy comparison by position for information reduction by gaze location multiplication.

Position	RMSE	Corr.	MAE
P1	0.992	0.356	0.799
P2	**0.775**	**0.697**	**0.653**
P3	1.053	0.303	0.810
P4	1.067	0.284	0.933

Note: P2 denotes standard position. The bold and underlined values are the most accurate.

**Table 12:** Accuracy comparison between human specialists and the proposed model.

Group/Model	RMSE	Corr.
Judges	1.278	0.592
Skaters	1.112	0.664
**Proposed model (No. 9)**	**0.775**	**0.697**

Note: The Judges and Skaters values are calculated from the experimental results in Part A. The bold and underlined values are the most accurate.

## Data Availability

The data are not publicly available due to breach of contract with the tracking company and with the subjects.
